# Quadrupled Hamstring Graft Diameter Adequacy in Anterior Cruciate Ligament Reconstruction Using Patient Anthropometry: A Prospective Cohort Study in Indian Males

**DOI:** 10.7759/cureus.15920

**Published:** 2021-06-25

**Authors:** Sunil Kumar, Harish Kumar, Prashant P Singh, Pranav Sharma, Amit K Rai Sharma, Mohit K Singh, Rajendra Kumar

**Affiliations:** 1 Orthopaedics, Uttar Pradesh University of Medical Sciences, Etawah, IND

**Keywords:** acl, anterior cruciate ligament, anthropometry, quadrupled hamstring graft diameter, height, autograft

## Abstract

Background and aim

The diameter of the graft used for the reconstruction of the anterior cruciate ligament (ACL) is an important determinant for the overall strength and future outcome of the operative procedure. Preoperative prediction of quadrupled hamstrings autograft (QHAG) diameter can prove to be of help in forecasting the need for augmentation or alternative grafts like quadriceps, bone-patellar tendon-bone autograft, and synthetic grafts. The relationship between the preoperatively assessed anthropometric parameters and the obtained quadrupled hamstrings graft diameter has not been extensively studied, especially in the population of Indian origin. This study aimed at investigating whether a correlation exists between the measured anthropometric parameters like age, weight, height, thigh circumference, and body mass index (BMI) and the intraoperatively obtained diameter of hamstring graft for ACL reconstruction in the study population of Indian male subjects.

Study design

A prospective cohort study conducted in a tertiary care center and teaching hospital in a district in central Uttar Pradesh, India.

Methods

The preoperative anthropometric data (age, height, weight, BMI, and thigh circumference of the injured side) of 73 Indian male subjects undergoing primary ACL reconstructive surgeries between May 2018 and August 2020 were prospectively collected, and their respective intraoperative QHAG diameters measured and recorded. Pearson’s correlation test was employed to determine the correlation between the preoperative demographic and anthropometric data and the obtained corresponding graft diameters. Simple linear regression was performed to obtain the graphical plots and determine the relationship between the dependent and independent variables. Of these, the variables showing significant association were subjected to stepwise linear regression to identify and exclude the confounder(s) and obtain the predicted equation.

Results

The study comprised 73 male participants. The study participants' mean age was found to be 33.7 years, mean height was 173.1 cm, mean weight was 71.2 kg, mean BMI was 23.7 kg/m^2^, mean thigh circumference was 50.4 cm, and the obtained mean graft diameter was 8.0 mm. A strongly positive correlation was observed between height and the graft diameter (r=0.940, P=0.000) and thigh circumference and the graft diameter (r=0.769, P=0.000). In contrast, weight showed a moderately positive correlation with the graft diameter (r=0.514, P=0.000). A very weakly positive correlation was observed between the BMI of the subjects and the obtained graft diameters (r=0.236, P=0.045). However, no correlation was observed between the age and the final graft diameters (r=0.140, P=0.238). Subsequent linear regression analysis indicates that only height (R^2^=0.883, P=0.000; strong) and the thigh circumference (R^2^=0.591, P=0.000; moderate) share a significant predictive value for the obtained QHAG. Both height and thigh circumference together were good predictors for graft diameter as determined by multiple regression (F (2,70)=272.372, P<0.001), with an R^2^ of 0.886.

Conclusion

Certain anthropometric parameters depict a positive correlation with the QHAG diameter and can assist in preoperative planning, predicting the possible harvested graft diameter and the need for alternative grafts or augmentation during ACL reconstructive surgeries.

## Introduction

Injury to the anterior cruciate ligament (ACL) is one of the most common athletic injuries encountered globally [1]. It is among the most common injuries around the knee [2]. The overall incidence of ACL injuries has been reported to be considerably higher in males than females [3]. Owing to its high incidence, ACL is the most frequently reconstructed ligament of the knee [4]. Among the variety of graft choices available for ACL reconstruction like hamstring tendon (HT) autograft, bone-patellar tendon-bone graft (BPTB), and allograft, HT autografts (semitendinosus and gracilis) have emerged to become the graft of choice (45-89% of the study population) for most orthopedic and knee surgeons, followed by BPTB graft (2-41%) and allograft (2-17%) [5]. Most surgeons prefer single-bundle reconstruction as the technique of choice for ACL reconstruction [5]. This progressive inclination toward HT autograft is well justified by the associated donor-site morbidity, higher risk of anterior knee pain, and loss of knee extension with BPTB autografts as compared with HT autografts, as has also been included in the Cochrane recommendations, even though both HT and BPTB autografts have comparable tensile properties, stiffness, functional outcomes, and knee stability [6-11].

The graft used most often, i.e., the HT autograft, utilizes the semitendinosus and gracilis tendons, derived from the affected side, doubled up twice onto themselves to create a four-strand graft (the quadrupled hamstring autograft). It has been reported that the tensile strength of the graft used increases significantly with an increase in the graft diameter [12].

Several studies have recommended the use of grafts with diameters equal to or greater than 8 mm, and that reconstruction with grafts lesser than 8 mm diameter has a higher risk of graft failures, poorer functional outcomes, and higher revision rates [13-16]. Owing to the high inter-individual variability in the size of HTs, the diameter of the derived graft varies and is often unpredictable. Preoperative prediction of the adequacy of the HT graft harvested during the surgery can prove to be of assistance in operative planning and help guide the surgeon regarding the need for augmentation or alternative grafts or fixation techniques, and subsequently counsel the patient regarding the possible graft choices and the functional outcome. It is, therefore, imperative to devise methods to predict the potential size of quadrupled hamstring autograft to be harvested intraoperatively. Several studies have attempted to address this by testing the relationship between various anthropometric parameters and the graft diameter, but no consensus has been achieved [16-24]. Moreover, only a few studies have been undertaken to study this relationship in the Asian population, let alone in India.

This prospective study aimed to determine whether any correlation exists between the various demographic and anthropometric parameters like age, weight, height, body mass index (BMI), and thigh circumference, and the harvested quadrupled hamstring autograft intraoperatively in ACL reconstruction among male subjects of Indian origin. We hypothesized that no such correlation exists between the dependent and the independent variables to analyze whether the harvested graft size can be preoperatively predicted using anthropometric measurements in our study population.

## Materials and methods

The study was conducted in a tertiary care center and teaching hospital in a district in central Uttar Pradesh, India. Approval of the study was acquired from the Institutional Ethics Committee, Uttar Pradesh University of Medical Sciences (Reference Number 125/2018). We used a prospective cohort study design to study the correlation of demographic and anthropometric data of subjects with the quadrupled hamstring autograft diameter in the ACL reconstruction. The enrollment of study subjects was done between May 2018 and August 2020 with appropriate written and informed consent.

Study participants

Population Under Study

All patients who present with a symptomatic acute ACL injury or a chronic ACL deficiency (categorized on the basis of time elapsed from injury till presentation) [25]. 

 

Inclusion Criteria

Male patients aged 17 years or more, presenting with an acute ACL injury who remained symptomatic after adequate rehabilitation (for three to six months, tailored according to patient's vocational needs and demands), and patients with chronic ACL deficiency planned for primary ACL reconstruction using anatomical single-bundle quadruple HT autograft.

 

Exclusion Criteria

Females; revision ACL reconstruction surgeries; patients undergoing ACL reconstruction using grafts other than HTs; double-bundle hamstring graft reconstructions; ACL injuries associated with other significant complete ligamentous injuries; ACL injuries with associated meniscal injuries that required suturing; acute ACL injury with tibial spine avulsion.

Study design

**Figure 1:**
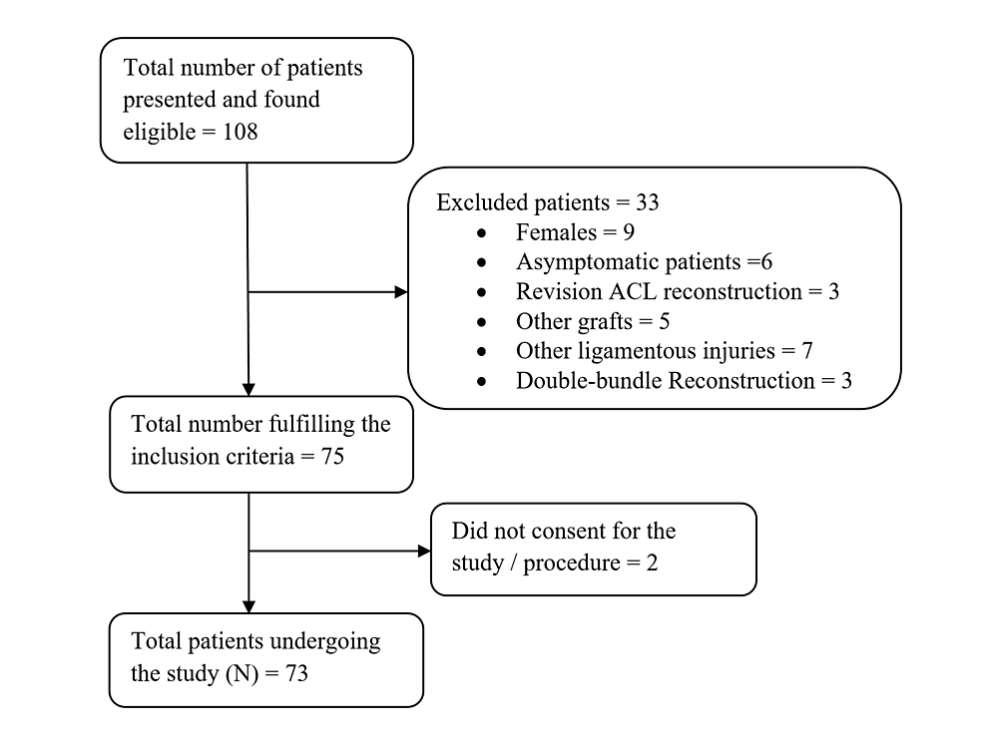
Procedure adopted for enrolling study participants N: total number of study participants; ACL: anterior cruciate ligament.

We adopted a purposive sampling strategy to obtain the sample size. From a sampling frame of 108 eligible patients who presented during the study duration and after exclusion of the subjects who did not fulfill the inclusion criteria, a total of 73 patients consented to the procedure and were enrolled to participate in the study (N=73) (Figure 1).

The study procedure and its significance were explained in detail to the study participants. Written and informed consent was obtained. The demographic profile, such as age and the anthropometric data, including weight, height, BMI, and thigh circumference, were obtained prospectively from the study participants before undergoing arthroscopic ACL reconstruction using quadrupled hamstring autograft. Blinding was achieved by ensuring that the surgeon, who himself is not involved in the operative procedure, collected the data for all the study subjects. Weight (in kg) and height (in cm) were recorded using the same weighing scale and stadiometer, respectively, for all the patients. The thigh circumference (in cm) of the affected side was measured using the same nonstretchable tape at a point 15 cm proximal to the superior pole of the patella, with the patient in supine position and the hip and the knee in full extension. The surgery was performed by the same certified orthopedic surgeon, trained and experienced in arthroscopic knee procedures, and both the semitendinosus and the gracilis hamstring grafts harvested using the same technique in all the patients. A standard 4-cm-long incision anteromedially over the proximal tibia was used to expose the pes anserinus for harvesting the hamstrings graft after releasing the tendons with an open-end tendon stripper. After separating muscle fibers from the tendons and trimming the tendons, a whipstitch was placed at both ends of each tendon with a nonabsorbable polyester suture. Then a four-strand graft was made by looping the derived tendons onto themselves. The final graft diameter measurement was then obtained by passing the graft through sizing cylinders (in increments of 0.5 mm) of the ACL reconstruction measurement guide (Arthrex). The diameter of the smallest calibrated sizing cylinder through which the entire graft could pass through smoothly was considered the final graft diameter. The operating surgeon, who was blinded from the anthropometric data, recorded the graft diameter intraoperatively. Single-bundle anatomic ACL reconstruction procedure was then carried out using standard techniques. Tightrope and interference screws were used as the femoral and tibial end fixation techniques, respectively, and these were kept similar in all the subjects. The relationship between the preoperatively measured anthropometric variables with the graft diameter obtained during surgery was then analyzed. 

Statistical analysis

We calculated the descriptive statistics of the various variables to describe the demographic profile of the population under study. The normality of the data distribution was ascertained using tests of normality. Pearson’s correlation test was used to determine whether there exists any correlation between anthropometric measurements (independent variables) and the obtained size of the quadrupled hamstrings autograft (dependent variable). The bivariate analysis further identified variables associated with graft diameter at a significance level of P<0.05. From this, the variables found to be associated at a P-value of <0.05 were further included in a stepwise linear regression to arrive at the predicted equation. Additionally, the potential confounder(s) were identified and excluded from the equation. A P-value of less than 0.05 (<5%) was considered statistically significant at a 95% confidence interval. All the outcome analyses were performed using the SPSS version 24.0 (IBM, Armonk, NY, USA).

## Results

A total of 73 male patients participated in the study. The means of the predictor and outcome variables, the anthropometric data, and the demographic profile of the study population are depicted in Table 1.

**Table 1:** Characteristics of the study participants (N=73) N: total number of study participants; SD: standard deviation; BMI: body mass index.

Variable	Mean ± SD	Range
Age (years)	33.7±11.2	17-57
Duration since injured (weeks)	15.1±7.2	4-32
Height (cm)	173.1 ±5.3	160.0-183.4
Weight (kg)	71.2±13.1	42.1-93.4
BMI (kg/m^2^)	23.7±3.9	15.5-33.1
Thigh circumference (cm)	50.4±6.8	39.1-64.7
Graft diameter (mm)	8.0±0.8	6.5-9.5

The analysis of preoperatively recorded data showed that the mean age of the patients at presentation was 33.7±11.2 years, the mean duration since injury was 15.1±7.2 weeks, the mean height of the study subjects was 173.1±5.3 cm, the mean weight was 71.2±13.1 kg, the mean BMI was 23.7±3.9 kg/m^2^, and the mean thigh circumference was 50.4±6.8 cm. Out of the 73 patients, 30 (41.09%) had sustained an injury to their left knee, whereas 43 (58.9%) had their right knee injured. Road traffic accidents (n=33, 45.2%) were the most common mode of injury, followed by sports and athletics-related events (n=27, 37%) (Table 2).

**Table 2:** Distribution of the mode of injury and the injured side among study participants N: total number of study participants.

Variables		Frequency (n)	Percentage
Mode of Injury	Fall from stairs	4	5.5
	Fall on ground	5	6.8
	Road traffic accident	33	45.2
	Sports injury	27	37.0
	Twisting injury	4	5.5
	Total (N)	73	100.0
Side of limb involved	Left	30	41.09
	Right	43	58.9
	Total (N)	73	100.0

The mean intraoperative diameter of the final quadrupled hamstrings graft was found to be 8.0±0.8 mm. Out of the total participants, 45 (61.6 %) had a graft diameter of 8 mm or more, the most frequent size being 8 mm (23.3%, n=17) (Table 3).

**Table 3:** Frequency distribution of graft sizes (N=73) N: total number of study participants.

Graft diameter (cm)	Frequency	Percentage	Cumulative percentage
6.5	6	8.2	8.2
7.0	10	13.7	21.9
7.5	12	16.4	38.4
8.0	17	23.3	61.6
8.5	13	17.8	79.5
9.0	10	13.7	93.2
9.5	5	6.8	100.0
Total	73	100.0	100.0

Computation and analysis of the Pearson’s correlation coefficient revealed a strongly positive correlation of the patients’ height (r=0.940, P=0.000) and thigh circumference (r=0.769, P=0.000) with the graft diameter, a moderately positive correlation between patients’ weight (r=0.514, P=0.000) and the graft diameter, and a very faintly positive correlation between patients’ BMI (r=0.236, P=0.045) and the graft diameter. No significant correlation was obtained between the patients’ age (r=0.140, P=0.238) and the quadrupled hamstring graft diameter harvested (Table 4).

**Table 4:** Distribution and correlation of anthropometric profiles with graft diameter (Pearson's correlation test applied) SD: standard deviation; BMI: body mass index.

Variables	r-Value	P-value
Age	0.140	0.238
Height	0.940	0.000
Weight	0.514	0.000
BMI	0.236	0.045
Thigh circumference	0.769	0.000

**Figure 2:**
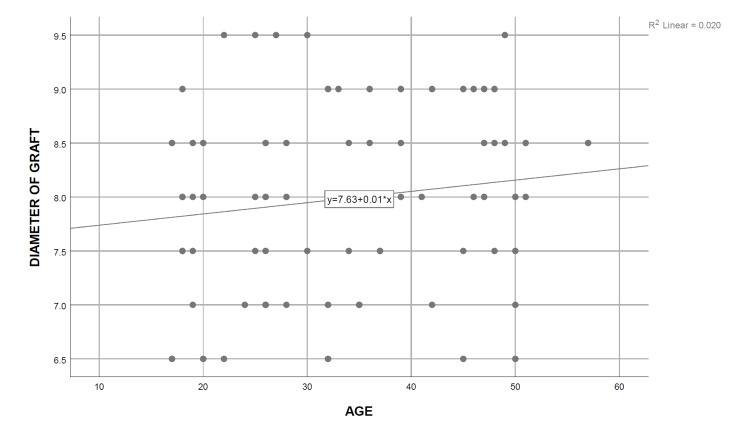
Curve estimate of the simple linear regression between the graft diameter and age

**Figure 3:**
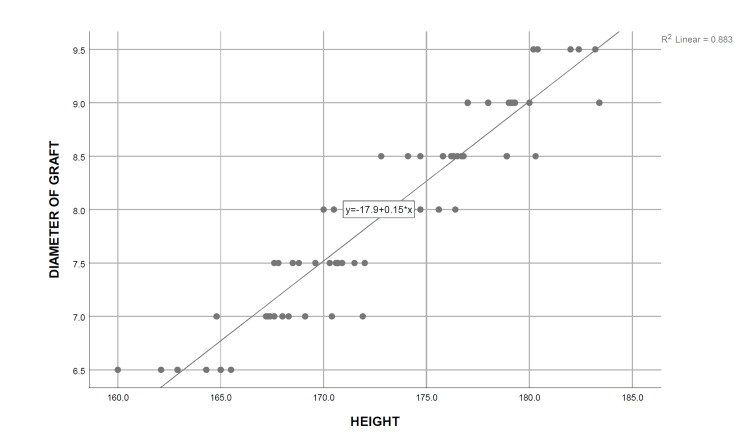
Curve estimate of the simple linear regression between the graft diameter and height

**Figure 4:**
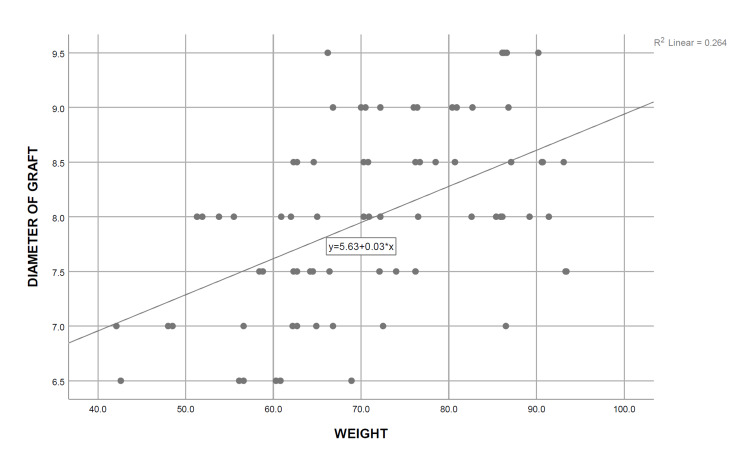
Curve estimate of the simple linear regression between the graft diameter and weight

**Figure 5:**
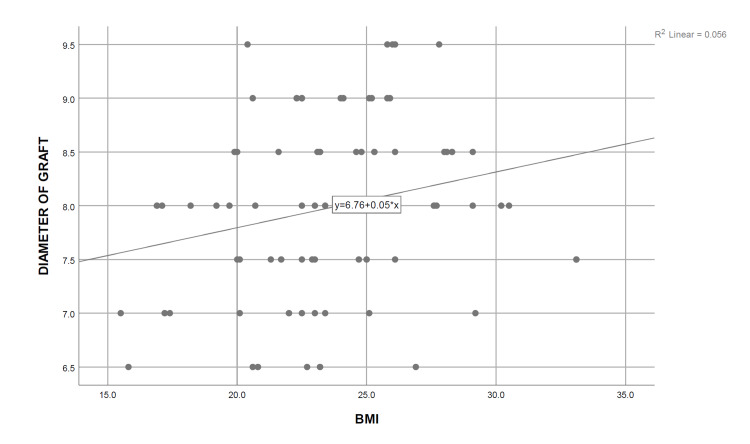
Curve estimate of the simple linear regression between the graft diameter and BMI BMI: body mass index

**Figure 6:**
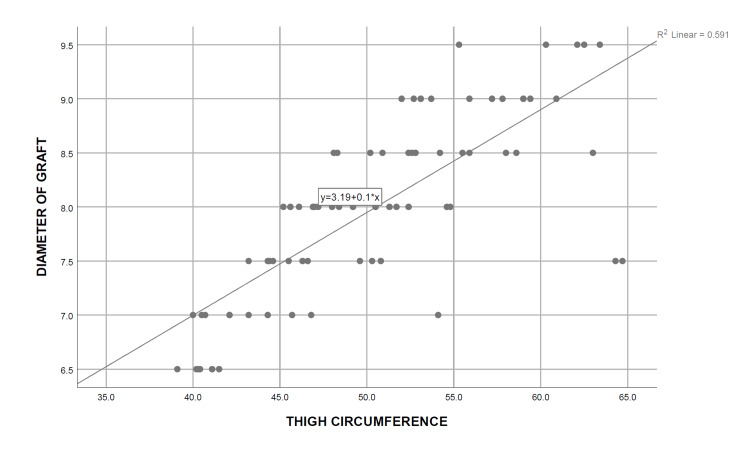
Curve estimate of the simple linear regression between the graft diameter and thigh circumference

A simple linear regression analysis was carried out, and the coefficient of determination was calculated for each independent demographic and anthropometric variable, keeping the obtained graft diameter as the dependent variable. It was determined that only height (R^2^=0.883, P=0.000), thigh circumference (R^2^=0.591, P=0.000), and weight (R^2^=0.264, P=0.000) are significant predictors of the final graft diameter, the strongest association being with the patients’ height. On the contrary, no significant relationship was observed between the age of the patients (R^2^=0.020, P=0.238) with the intraoperatively obtained graft diameter, whereas BMI (R^2^=0.056, P=0.045) showed only a weak association with the graft diameter (Table 5) (Figures 2-6). Multiple regression analysis was performed to determine the best linear combination of independent variables for predicting the final graft diameter. Weight and BMI were found to be the potential confounders on stepwise multiple regression models. Subsequently, the graft diameter was predicted based on the height and the thigh circumference. A significant regression equation was found, (F (2,70)=272.372, P<0.001), with an R^2^ of 0.886. Participants’ predicted graft diameter is equal to -16.574+0.011 (thigh circumference)+0.139 (height), where both height and thigh circumference were measured in centimeters. Participants’ graft diameter increased by 0.011 mm with each centimeter increase in thigh circumference and by 0.139 mm with each centimeter increase in height of the subjects, respectively. Both height and thigh circumference were significant predictors of graft diameter.

**Table 5:** Results of the simple linear regression analysis between the graft diameter and the anthropometric profiles R^2^: coefficient of determination; BMI: body mass index.

Variables	R^2^	P-value
Age	0.020	0.238
Height	0.883	0.000
Weight	0.264	0.000
BMI	0.056	0.045
Thigh circumference	0.591	0.000

 

## Discussion

Quadrupled hamstring autograft is the most preferred graft option for ACL reconstruction [5]. The diameter of graft harvested and utilized for reconstruction has a significant relationship with the strength of the graft [12]. The knowledge of the intraoperative diameter of the graft to be harvested can help predict the acceptability of the graft and its future implications like the risk of failure and need for revision surgery and, hence, can guide the surgeon with operative planning.

Upon analysis of the study outcomes based on our study population of 73 Indian males undergoing primary ACL reconstruction, we observed a positive correlation between the height, thigh circumference, weight, and the BMI of the study subjects, with the diameter of harvested HT autograft. Of these variables, height and thigh circumference were found to have a strong correlation, weight was only moderately correlated, and BMI had a poor correlation with the obtained graft diameter. Patients' age showed no correlation with the graft diameter. Further analysis using linear regression showed that only height (R^2^=0.883, P=0.000), thigh circumference (R^2^=0.591, P=0.000), and weight (R^2^=0.264, P=0.000) were significantly associated with the size of the graft diameter, with the height being the most strongly related. This strength of the relationship was justified to be strong, as up to 88.3% of the variance in the harvested graft diameter could be attributed to height, and thigh circumference could explain up to 59.1% of the variability in the graft diameter. In contrast, only 26.4% and 5.6% of the graft diameter variance could be attributed to weight and BMI, respectively. Age had no statistically significant relationship with the harvested graft diameter.

Furthermore, on analyzing multiple regression models, the relationship with weight and BMI was also disregarded due to their confounding effect on the outcome. Height was shown to have the most significant predictive value for the harvested graft diameter. It was observed that thigh circumference alone had only a moderately significant relationship with the dependent variable (graft diameter); however, together with height, they showed a positive association with the dependent variable and a significant predictive value for harvested graft diameter, with an R^2^ of 0.886. This indicates that 88.6% of the variance in the graft diameter could be explained by the model having the combination of these two variables. The combination of both height and thigh circumference together significantly predicted the final graft diameter.

Among all the independent variables, height was determined to be the most significant predictor of harvested quadrupled hamstring graft diameter in ACL reconstruction. This stands in corroboration with multiple studies conducted previously [18,19,21,26,27]. In a study conducted by Tuman et al., it was determined that height is the best predictor for HT graft diameter in subjects of both genders, although the correlation was the strongest in females [18]. They observed that the graft diameter was related to height, mass, age, and gender but had no relation with the BMI of the subjects. Based on this, Treme et al. further analyzed these outcomes in a separate study and concluded that graft diameter is most strongly correlated with weight and thigh circumference of the subjects, whereas there is no correlation of graft diameter with the age of the subjects [20]. Ma et al. reported height to be a specific predictor of graft diameter solely in males [19].

A significant correlation between the thigh circumference and the graft diameter of the subjects was observed in the preliminary analysis in our study, but upon further investigation using linear and multiple regressions, only a moderate predictive power for the graft diameter could be ascertained to the measured thigh circumference alone. This is in contrast to the findings of Treme et al. and Asif et al., who documented thigh circumference to be a significant predictor of the HT graft diameter [17,20]. Gupta et al. also showed a weak correlation between the thigh circumference and the graft diameter [28].

We observed a weak correlation between the weight of the subjects and the size of grafts; however, upon multiple regression analysis, it was found to act as a confounder. Weight has also been reported to have no significant association with the graft diameter in other studies previously [19,21]. Whereas, in contrast, several authors have reported the graft diameter being strongly related to the weight of the subjects [18,20,26,27]. These differences in observations can possibly be attributed to the varying degrees of body fat in different individuals, which contributes to the weight but not the muscle mass of the individuals.

It has previously been shown that the graft diameter is not related to BMI, as demonstrated in multiple separate studies [18,21, 23,24,26,27]. Our analysis exhibits a similar finding regarding BMI. One possible cause for this particular finding could be that BMI takes into account the whole body mass and not just the lean muscle mass. Similarly, our study found no statistically significant correlation between age and graft diameter, as has also been demonstrated before [18-21,24,26]. Although a study by Moghamis et al., in contrast, reported a moderately significant positive correlation between age and the harvested graft diameter, there was no correlation with height or BMI [23].

Studies involving both genders have demonstrated that females have a significantly smaller graft diameter than men [18,26]. Treme et al., in their research, exhibited that ipsilateral thigh circumference remains the sole important predictor for graft diameter in females and that use of gender-specific recommendations provides better accuracy in predictions and identification of individuals at risk of insufficient graft diameter [20]. In another similar study, Stergios et al. could not derive any statistically significant predictor for graft diameter in female patients [29]. Based on this premise, female subjects requiring ACL reconstruction had been excluded from our study, and only male subjects were enrolled.

Our findings strongly correlate with the similar studies undertaken previously on subjects of Indian origin, especially in regard to height having the strongest correlation and being the most significant predictor [17,22,24,28]. Asif et al. concluded that the height and the thigh circumference of the subjects are significantly related to the hamstring graft diameter, whereas BMI has no influence on the same [17]. Challa et al. also reported height to be of most predictive influence on graft diameter for both males and females of their Indian study population but found no other variable to correlate with the graft diameter [24]. Similar outcomes were observed by Goyal et al., where they found only height to be most strongly related to the harvested graft diameter, although the strength of association between the two variables was only moderate [22]. Another study that supports the above findings is by Gupta et al., where height was found to be strongly correlated with the graft diameter, weight was moderately correlated, whereas thigh circumference was only weakly correlated with the intraoperatively observed graft diameter [28]. In addition to the above findings, a key conclusion in our study was the demonstration of superior predictive value for graft diameter when using a combination of height and thigh circumference.

Limitations

This study does not take into account the possible differences in measuring the thigh circumference due to the position of the patient (supine or standing) and the site of measurement (15 cm from the superior pole of patella or mid-point between the mid-inguinal crease and the superior border of patella) and its possible effects on relationship with the final predicted graft diameter. Moreover, we measured the weight (and the BMI) of the study subjects, which do not address the varying body fat percentages in different individuals and can be a potential source of discrepancy. Future studies with measurements of lean body mass instead can be of help in this regard. Also, studies with larger sample sizes are needed to ascertain the definitive relationship between the variables under study.

## Conclusions

Preoperative measurements of certain anthropometric parameters can prove to be a valuable tool for predicting the adequacy of intraoperatively harvested graft diameter, the need for augmentation or alternative grafts, and the risk of failure or the graft used. This can further help reduce the operation time, postoperative pain, and scar if an alternative graft is chosen due to inadequacy.

Height remains the most consistent parameter among the variables studied for the prediction of the harvested quadrupled hamstrings autograft diameter, even in the study population of Indian males. The predictive value was seen to improve further when thigh circumference and height are used in combination.
